# MIF functional polymorphisms are associated with acute GVHD progression and steroid-refractoriness

**DOI:** 10.3389/fimmu.2025.1504976

**Published:** 2025-04-30

**Authors:** Aviran Aharon, Batia Avni, Daniel Louzoun, Shlomo Elias, Polina Stepensky, Ron Ram, Tsila Zuckerman, Roberto Meza-Romero, Arthur A. Vandenbark, Sigal Grisariu, Gil Benedek

**Affiliations:** ^1^ Hebrew University-Hadassah Faculty of Medicine, Jerusalem, Israel; ^2^ Department of Bone Marrow Transplantation and Cancer Immunotherapy, Faculty of Medicine, Hadassah Medical Center, Hebrew University of Jerusalem, Jerusalem, Israel; ^3^ Bone Marrow Transplantation and Cellular Therapy Unit, Tel Aviv Medical Center and Faculty of Medicine, Tel Aviv University, Tel Aviv, Israel; ^4^ Department of Hematology and Bone Marrow Transplantation, Rambam Health Care Campus, Haifa, Israel; ^5^ The Ruth and Bruce Rappaport Faculty of Medicine, Technion, Haifa, Israel; ^6^ Department of Neurology, Oregon Health & Science University, Portland, OR, United States; ^7^ Neuroimmunology Research, VA Portland Health Care System, Portland, OR, United States; ^8^ Department of Molecular Microbiology & Immunology, Oregon Health & Science University, Portland, OR, United States; ^9^ Tissue Typing and Immunogenetics Unit, Department of Genetics, Hadassah-Hebrew University Medical Center, Jerusalem, Israel

**Keywords:** MIF, acute GVHD, steroid-refractory, HSCT, polymorphism

## Abstract

**Introduction:**

Approximately 50% of allogeneic hematopoietic stem cell transplantation (HSCT) Q6 recipients develop graft versus host disease (GVHD). Glucocorticoids (GC) are the first line of treatment for both acute and chronic GVHD. Failure to respond to GC [steroid-refractory (SR)] encompasses a very poor outcome with high mortality. Macrophage migration inhibitory factor (MIF) is released during transplantation and triggers enhanced and prolonged immune reactions. Persistently elevated levels of MIF have been shown to override both endogenous and exogenous antiinflammatory effects of GC.

**Methods:**

Two functional polymorphisms in the MIF gene, a −794 CATT5–8 microsatellite repeat and a −173 G/C single-nucleotide polymorphism, were analyzed in 86 patients who underwent allogeneic HSCT. We also measured MIF serum levels at different time points before and after HSCT.

**Results:**

Frequencies of MIF high-expression -794 CATT7 containing genotypes were increased in patients with grade III-IV acute GVHD (aGVHD) (36.8%) compared with patients that did not develop aGVHD (5.8%) and patients with grade II aGVHD (0%), (p=0.0019, 0.0080 respectively). We also demonstrated that the frequencies of the MIF-794 CATT7 and -173 C containing genotypes, were significantly associated with steroid-refractory aGVHD (46.6%, 60% respectively) compared to steroid-responsive aGVHD (0%, 5.3% respectively), (p=0.0011, P=0.0007 respectively). We further showed that MIF circulating levels preceded onset of severe aGVHD.

**Discussion:**

Our findings suggest that genetically controlled high expression MIF genotypes are associated with aGVHD worsening and could serve as a biomarker enhancing identification and treatment of steroid-refractory disease.

## Introduction

Allogeneic hematopoietic stem cell transplantation (HSCT) has become the treatment of choice for a wide variety of hematologic malignancies and non-malignant disorders (1). Increasing numbers of HSCT are being performed every year and their indications have expanded in the recent years. However, graft-versus-host disease (GVHD) remains a significant challenge to the broader use of allogeneic HSCT. Once established, managing it becomes exceedingly difficult. Although GVHD prophylaxes exist, they come with the tradeoff of increased risk of relapse, rejection, or delayed immune recovery. Consequently, although significant progress has been made, there is no universally effective strategy for preventing or treating GVHD, particularly steroid-refractory disease. This immune-mediated condition is the most serious complication following HSCT, affecting approximately half of all recipients and causing immune dysregulation and organ dysfunction (2–5).

Several risk factors for developing GVHD were identified including: histocompatibility disparity, donor and recipient age, donor and recipient sex disparity (female donor to male recipient), intensity of conditioning regimen, use of total body irradiation (TBI), and stem cells source (2–5). Classically, the pathophysiology of acute GVHD has three phases: The afferent phase, in which an exaggerated inflammatory response leads to activation of antigen presenting cells (APCs). This phase is followed by the efferent phase, which is characterized by donor T-cell trafficking and expansion. Lastly, the effector phase, in which effector cells cause end-organ damage (6, 7).

Systemic high dose Glucocorticoids (GC) is the standard first line therapy for acute and chronic GVHD (aGVHD and cGVHD, respectively). GC have broad anti-inflammatory and regulatory effects on most of the immune cells: They reduce production of pro-inflammatory cytokines, decrease cell migration to sites of inflammation and promote apoptosis of activated cells (8, 9). In addition, GC were shown to promote regulatory T cells and anti-inflammatory macrophages (10, 11). However, therapeutic response ranges from approximately 60% in patients with grade II aGVHD to 30 to 40% in patients with grade IV disease. More than 70% of patients suffering from cGVHD will need further treatment due to the toxicity and limited efficacy of initial therapies. In both patient groups, GC refractoriness encompasses a dismal prognosis (12–16).

Thus, identifying patients that might develop GVHD and might not respond to GC treatment (steroid-refractory (SR)) and initiating early enough alternative treatments might prevent worsening of the disease and spare steroid toxicity.

Macrophage migration inhibitory factor (MIF) is a key mediator of many inflammatory diseases such as septic shock, rheumatoid arthritis, atherosclerosis and multiple sclerosis (17–23). It was reported that MIF primarily drives disease progression by promoting inflammatory cell recruitment, preventing apoptosis of activated cells and amplifying the secretion of pro-inflammatory cytokines (24, 25). MIF is secreted from various cells types such as, dendritic cells, macrophages, T cells and endothelial cells (26, 27). MIF engagement of its receptor, CD74, leads to the recruitment and activation of CD44 and CXCR2/4/7 to initiate signaling pathways necessary for mitogen-activated protein kinase (MAPK) activation, which leads to cell motility, migration and survival (28–30). MIF has a central role in counter-regulating glucocorticoid action. Increased inflammation in turn triggers rapid production of endogenous GC that eventually resolve inflammation and reduce MIF levels (31). However, chronically high MIF levels override the GC effects (32–34).

Interestingly, resistance to GC therapy is also common and occurs in up to 30% of subjects suffering from arthritis, inflammatory bowel disease and asthma. Additional studies have demonstrated that these patients express increased levels of MIF compared with GC responsive patients (35–39).

MIF expression is genetically regulated by two functional polymorphisms located in the *MIF* gene: alleles of the −794 CATT_5–8_ microsatellite repeats and the −173 G/C single nucleotide polymorphism (SNP), which have been reported to modulate *MIF* promoter activity and to correlate with MIF expression levels. *MIF* promoter activity is proportional to increased numbers of the CATT repeats at position −794, whereas the −173 C allele may be associated with increased *MIF* promoter activity by its linkage disequilibrium with the high-expression of the −794 CATT_7_ variant (27, 40).

Only a handful of studies have examined the role of MIF in GVHD. Their findings have shown that MIF is increased significantly in serum at the onset of aGVHD compared with levels before allogeneic HSCT. They also demonstrated that mice that received bone marrow (BM) cells from MIF knockout (KO) mice exhibited a milder form of aGVHD compared with mice that received BM cells from wild type mice (41, 42). Furthermore, Chang et al. demonstrated that patients receiving cells from donors who carry the MIF -173C allele (which correlates with high MIF expression) were at higher risk of developing chronic GVHD. However, in the same report it was suggested that recipients carrying the -173C allele have better overall survival rates and lower risk of relapse (43). Taken together, this implies that MIF might have a pivotal role as disease severity modifiers in patients suffering from aGVHD and moreover in those resistant to GC treatment.

In this study, we conducted a comprehensive evaluation of the role of MIF in patients undergoing HSCT, with a specific focus on its involvement in those who developed aGVHD and SR aGVHD. This involved measuring analyzing the frequencies of functional MIF promoter variants as well as measuring MIF serum levels, before and after HSCT.

## Material and methods

### Study participants

The study cohort included patients above the age of 18 years old who underwent allogeneic HSCT between March 2020 and February 2024, at three different transplantation centers in Israel: The Department of Bone Marrow Transplantation at Hadassah Medical Center in Jerusalem, Sourasky Medical Center in Tel Aviv, and Rambam Medical Center in Haifa. After providing informed consent, blood samples were collected from the study participants during the week preceding the initiation of the conditioning regimen. Additional blood samples were taken at 14, 30, and 90 days post-HSCT.

Acute GVHD was graded according to Mount Sinai Acute GVHD International Consortium (MAGIC) criteria for acute GVHD (44) and the EBMT-NIH-CIBMTR Task Force position statement on standardized terminology & guidance for graft-versus-host disease assessment (45). Acute GVHD steroid refractoriness was defined as: progression in any organ within 3 days of therapy onset with ≥2 mg/kg/day of prednisone equivalent, failure to improve within 5 days of treatment initiation or incomplete response after more than 28 days (45). Myeloablative regimens included: TBI ≥ 500 cGy as a single fraction or ≥ 800cGy if fractionated, total busulfan ≥ 9mg/kg, total melphalan ≥ 150mg/m^2^, total Thiotepa ≥ 10mg/kg and total treosulfan ≥36g/m^2^. Any other conditioning regimen utilized was categorized as reduced-intensity conditioning (RIC) regimen.

This study complies with the Declaration of Helsinki. The relevant Institutional Review Boards approved the study, (number 0286-18-HMO) and all subjects gave their signed informed consent to participate.

### MIF-173G/C genotyping

High molecular weight DNA was extracted from blood samples that were drawn before the conditioning, using the MagLEAD DNA isolation kit with the automatic device MagLEAD g12c (PSS).

A 366-bp fragment of DNA containing the MIF −173 polymorphism (rs755622) was amplified as described before (46). Briefly, PCR was performed using 200 ng of DNA, 0.5 mM of primers: forward primer (5’-ACTAAGAAAGACCCGAGGC-3’), reverse primer (5’-GGGGCACGTTGGTGTTTAC-3’), and REDExtract-N-Amp PCR Ready Mix (#R4775; Sigma Aldrich). The PCR product was digested overnight using AluI restriction site and was cut into two fragments resulting in a 98- and a 268-bp band, while the C allele contains two AluI restriction sites and was cut into three fragments, resulting in 205-, 98-, and 63-bp bands.

### MIF –794 CATT5–8 genotyping

The MIF -794 CATT5-8 microsatellite was analyzed by the methodology described by Sreih at al. (47). Briefly, MIF -794 CATT5-8 genotyping was carried out by PCR using a forward primer (5′- GCAGGAACCAATACCCATAGG-3′) and a FAM fluorescent reverse primer (FAM 5′-AATGGTAAACTCGGGGGAC-3′). Automated capillary electrophoresis on a DNA sequencer was performed on each sample and the -794 CATT5-8 repeat length was identified using Genotyper 3.2 software (Applied Biosystems). Although at least eight additional polymorphisms have been identified within the human MIF locus, these additional variants (all SNPs) are rare and have a low likelihood of functionality given their location in introns or within the 3′UTR (48).

### Analysis of plasma MIF levels by ELISA

Plasma was isolated from blood samples of 60 patients, from this cohort at different time points. Plasma MIF concentration was measured by the human MIF Quantikine ELISA kit (DMF008, R&D systems) according to the manufacturer’s instructions. The minimum detectable dose of human MIF using this kit ranges from 0.005-0.068 ng/ml (mean = 0.016ng/ml).

### Statistical analysis

According to average odds ratio for MIF association with disease progression and steroid resistance (OR=2-4) (49, 50), to obtain an effect size of W=0.6 with a statistical power of 80%, we aimed to recruit at least 85 subjects that underwent HSCT.

For MIF polymorphisms, Pearson’s χ2 test was used to analyze Hardy–Weinberg equilibrium. Genotype frequencies were compared using Fisher’s exact χ2 test. Multivariate logistic regression analysis was performed using NCSS software (version 24.0.3). MIF serum levels were compared using Mann-Whitney test. Tests with p<0.05 were considered significant.

## Results

### Patient characteristics

A total of 86 patients transplanted between January 2020 and March 2024, were included in the study. The median follow-up was 442 days post-transplantation (range 35-1318 days). The clinical characteristics and demographic data are summarized in **Table 1**. The study cohort included 39 females and 47 males with a median age of 57 (range 18-75) years old. The primary underlining disease was acute myeloid leukemia (AML) (61.6%). Most of the patients were transplanted from a matched unrelated donor (MUD) (48.8%). Most of the male patients were transplanted from a male donor (64%). Conditioning intensity was mainly myeloablative (MA) (60.4%).

**Table 1:** Patients and transplant characteristics.

Number of patients	N=86
Recipients Age (median, range)	57 (18-75)
Gender (females/males)	39 (45.3%)/47 (54.7%)
Diagnosis	
AML	53 (61.6%)
ALL	13 (15.2%)
MDS	11 (12.8%)
Other	9 (10.4%)
Age adjusted HCT comorbidity index	
Low	16 (18.6%)
Intermediate	43 (50.0%)
High	27 (31.4%)
Donors Age (median, range)	30 (19-68)
Recipient-donor sex mismatch (female to male)	16 (21.6%)
Donor type	
MRD	31 (36.0%)
MUD	42 (48.8%)
MMUD	7 (8.2%)
Haploidentical	6 (7.0%)
Pre-Transplant disease status	
Active malignant disease	13 (15.1%)
MDS (not applicable)	11 (12.8%)
CMV sera status (donor to recipient)	
Negative to negative	6 (7.0%)
Negative to positive	15 (17.4%)
Positive to negative	10 (11.6%)
Positive to positive	55 (64.0%)
Stem cell source	
PBSC	85 (98.8%)
BM	1 (1.2%)
Conditioning regimen intensity	
MAC	52 (60.4%)
RIC	34 (39.6%)
GVHD prophylaxis	
CSA-MTX	47 (54.7%)
CSA-MMF	33 (38.4%)
PTCY	6 (6.9%)
ATG	54 (62.8%)
Follow-up, days (median, range)	442 (35-1318)

AML, acute myeloid leukemia; ALL, acute lymphoblastic leukemia; MDS, myelodysplastic syndrome; MRD, matched related donor; MUD, matched unrelated donor; MMUD, mismatched unrelated donor; PBSC, peripheral blood stem cell; BM, bone marrow; MAC, myeloablative conditioning; RIC, reduced intensity conditioning; CSA, cyclosporine; MTX, methotrexate; MMF, mycophenolate mofetil; PTCY, post-transplantation cyclophosphamide; ATG, anti-thymocyte globulin; CMV, ctyomealovirus. Active disease=not in complete remission.

Forty-six patients developed aGVHD. Thirty-four patients developed aGVHD grade II- IV and of them, 15 (44%) were refractory to steroids (**Table 2**). In univariate analysis, none of the following variables (HLA matching, sex matching, conditioning intensity, ATG treatment, cytomegalovirus (CMV) sera-status, pre-transplant disease status or patient’s and donor’s age) were significantly associated with aGVHD.

**Table 2:** Characteristics of patients with aGVHD.

	N=86
aGVHD – all grades	46 (53.5%)
aGVHD grade	N=86
I	12 (14%)
II-IV	34 (39.5%)
III-IV	18 (21%)
aGVHD onset (days post HSCT) (median, range)	
	36 (12-302)
Steroid refractory	
All grades (N=46)	15 (32%)
Grade II-IV (N=34)	15 (44%)

### MIF promoter polymorphisms in aGVHD

The prevalence of the MIF -173G/C SNP and -794 CATT5-8 microsatellite polymorphisms were evaluated in the entire study cohort, from blood samples that were drawn before starting the conditioning regimen. First, we tested the Hardy-Weinberg equilibrium. No deviation from Hardy-Weinberg equilibrium was detected in this group (CATT5-8 p=0.94, G/C p=0.30). Next, we compared the frequencies of MIF polymorphisms between patients that did not develop aGVHD or developed grade I aGVHD, to patients that developed aGVHD grades II-IV. The frequencies of the MIF high-expression -794 CATT7 containing genotypes were significantly higher in patients that develop aGVHD grade II-IV compared with patients that did not develop aGVHD or developed grade I aGVHD (20.6% vs. 5.8% p=0.045 OR=4.23). In addition, we evaluated the MIF -173 G/C polymorphism, but did not observe any significant difference in the frequencies of the C allele genotypes (**Supplementary Table 1**).

**Figure 1:**
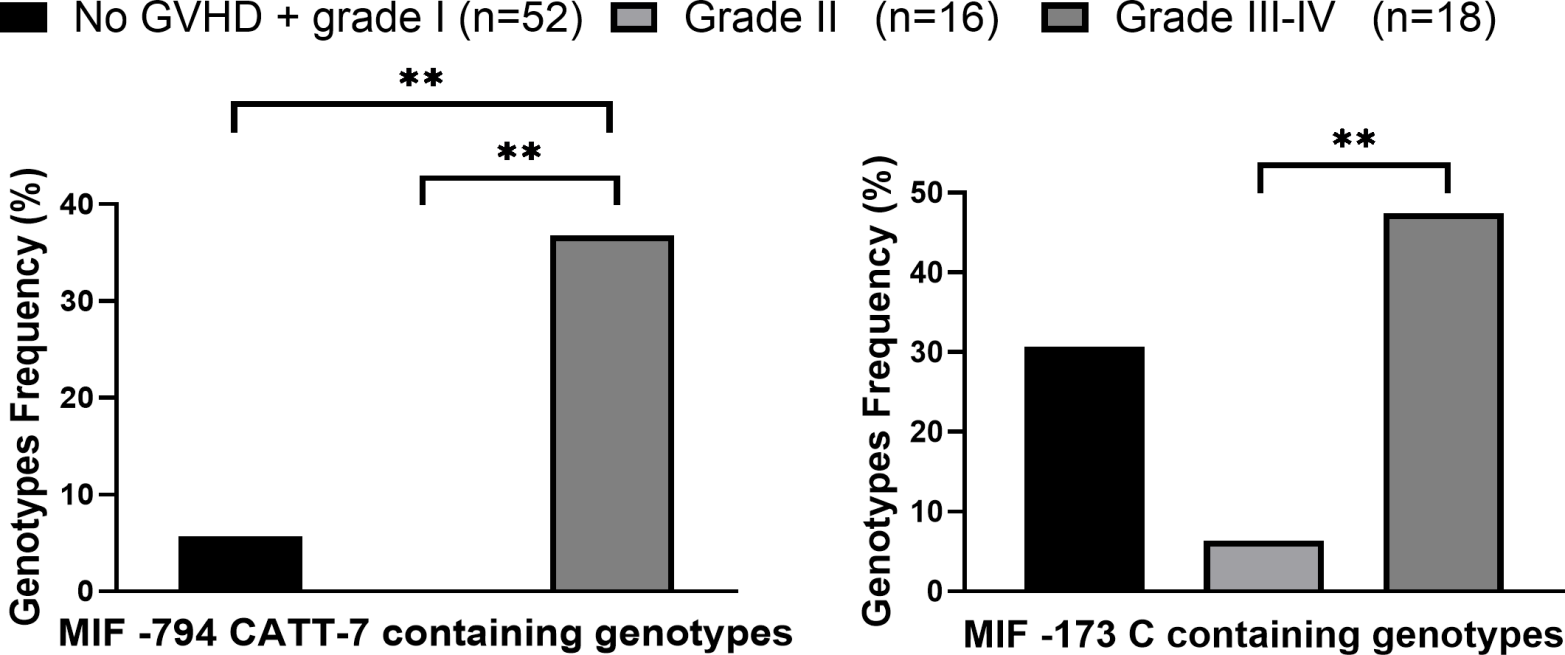
High expression MIF genotypes frequencies in patients who underwent HSCT. MIF promoter genotypes were evaluated in HSCT patients that did not develop aGVHD\ grade I aGVHD (*black*), patients that developed Grade II (*light gray*) and grade III-IV aGVHD (*dark gray*). P values were calculated by Fisher exact test. **p<0.01.

MIF expression was shown to affect the progression of different diseases (17, 27, 47). In order to evaluate whether MIF polymorphisms were associated with aGVHD severity, patients were divided according to disease severity: No or grade I aGVHD vs. grade II vs. grades III- IV. The frequencies of the high-expression CATT7 containing genotypes were significantly higher in patients that develop severe aGVHD compared with patients that developed grade II aGVHD (36.8% vs. 0%, p=0.0019 OR=10.4). These frequencies were also significantly higher when compared to patients with no or grade I aGVHD (36.8% vs. 5.8% p=0.0080 OR=10.2) as demonstrated in **Figure 1**; **Supplementary Table 2**. Interestingly, six of seven patients (86%) that developed aGVHD grade IV carried the CATT7 allele. There was no significant difference in the frequencies of the CATT7 containing genotypes between patients that did not develop aGVHD or developed grade I aGVHD, and patients that developed grade II aGVHD. The increased frequency of the CATT7 containing genotypes in patients who developed grades III-IV aGVHD, remained highly significant even after multivariate analysis that included the conditioning regimen and whether the patients were transplanted from a matched or mismatched donor (p=0.0008, OR=13.21). The association of MIF also remained significant after multivariate analysis that included the use of ATG and sex mismatch (p=0.0005, OR=18.26).

The frequencies of the MIF -173 C containing genotypes were significantly higher in patients with grades III-IV aGVHD compared with patients with grade II aGVHD 47.7% vs. 6.3% p=0.0078 OR=15.0) (**Figure 1**). Taken together, our data demonstrate that genetically regulated high MIF expression is associated with aGVHD severity.

### MIF polymorphisms in steroid-refractory aGVHD

**Figure 2:**
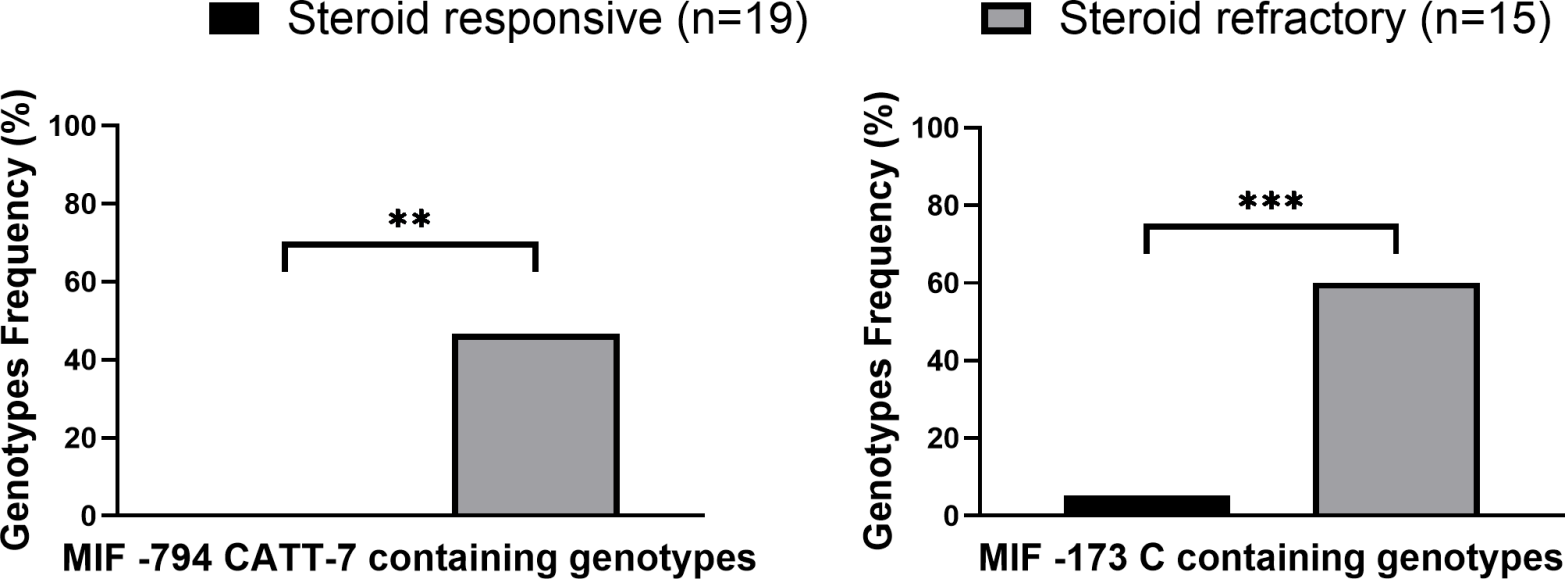
High expression MIF genotype frequencies in grade II-IV GVHD patients. MIF prompter genotypes in GVHD patients that responded (*black*) to steroid treatment or did not respond (*gray*) to steroid treatment. P values were calculated by Fisher exact test. **p<0.01. ***p<0.001.

In our cohort, 44% (15/34) of patients that developed modest to severe aGVHD (grades II-IV) did not respond to GC treatment. In accordance with MIF’s role as a GC regulator, the frequencies of -794 CATT7 containing genotypes and -173C containing genotypes were significantly higher in patients with steroid-refractory aGVHD compared with steroid-responsive disease (46.6% vs. 0% p=0.0011 OR=16.6 and 60.0% vs. 5.3% p=0.0007 OR=27.0, respectively) (**Figure 2**; **Supplementary Table 3**). These findings show that MIF polymorphisms are associated with steroid-refractory aGVHD.

### MIF serum levels in aGVHD

**Figure 3:**
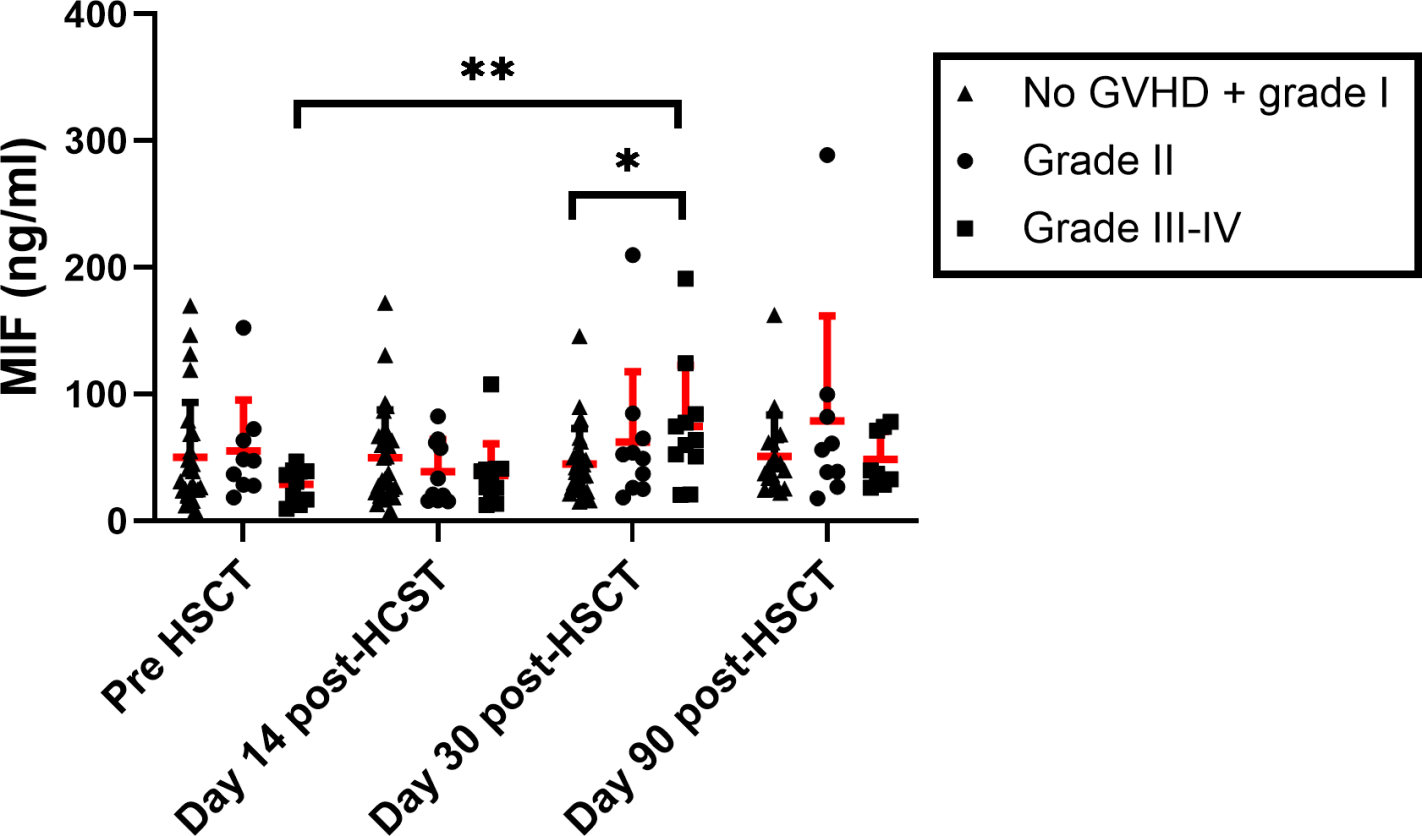
MIF serum levels in HSCT patients. MIF levels were evaluated in patients that received HSCT at 4 time points: pre-transplantation, 14, 30 and 90-days post-transplantation. The patient groups were categorized based on the severity of aGVHD (*triangle*- No aGVHD and grade I, *circle*- Grade II *square*- Grade III-IV). The means are depicted as red line with SEM. *p<0.05, **p<0.01 Mann-Whitney test.

It was previously demonstrated that MIF levels were upregulated during aGVHD onset (41, 42). We evaluated these findings in our patients’ cohort. MIF serum levels were evaluated in samples from 60 patients: 35 patients that did not develop aGVHD or developed grade I aGVHD and 25 patients that developed aGVHD (12 Grade II and 13 grade III-IV). There was no significant difference in MIF levels prior to conditioning and two weeks post-HSCT between patients that developed or did not develop aGVHD. However, at 30 days post-HSCT MIF plasma levels were significantly elevated in patients who developed aGVHD grade II-IV compared with patients that did not (68 ± 51 ng/ml vs. 44 ± 28 ng/ml, p=0.048). Furthermore, in patients with grade II-IV, MIF levels at 30 days post-HSCT were significantly higher compared to their pre-transplantation levels (67 ± 51 ng/ml vs. 41 ± 32 ng/ml, p=0.011) (**Supplementary Figure 1**). We next analyzed these data according to aGVHD severity. No significant differences were observed between patients with grade II aGVHD compared with patients with no aGVHD or grade I aGVHD. However, at 30 days post-HSCT, MIF serum levels were significantly higher in patients with grade III-IV aGVHD compared with patients that did not develop aGVHD or developed grade I aGVHD (74 ± 48 ng/ml vs. 44 ± 28 ng/ml, p=0.031). In addition, at 30 days post-HSCT, MIF levels were significantly higher in patients with grade III-IV aGVHD compared to their pre-transplantation levels. No difference was observed between samples, which were taken at day 90 post-HSCT (**Figure 3**). It is important to note that several of the patients with severe aGVHD (mainly those with grade IV) did not survive and were not sampled at this time point. We did not observe any significant difference in MIF serum levels between patients with aGVHD that responded to GC treatment compared to patients with steroid-refractory aGVHD.

**Figure 4:**
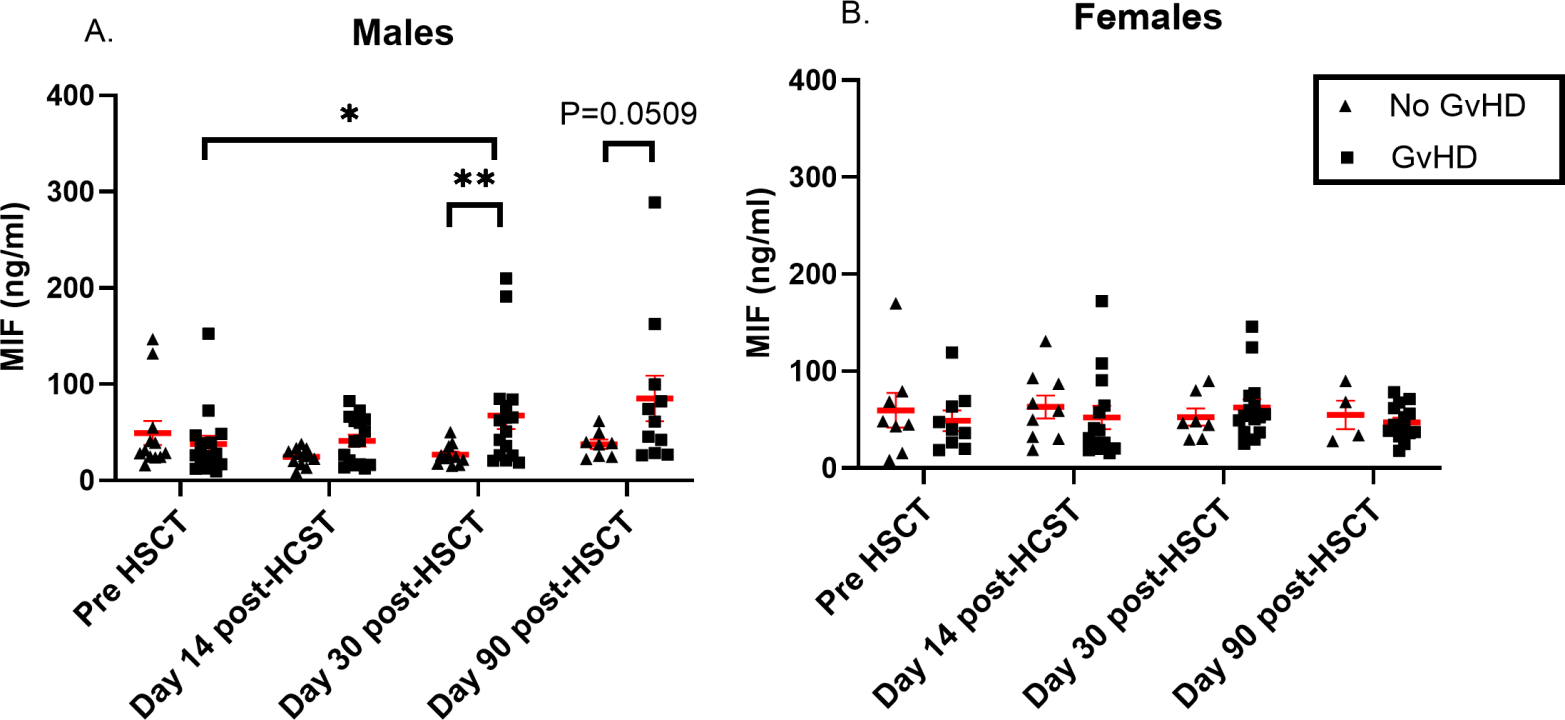
MIF serum levels in HSCT patients. MIF levels were evaluated in patients that received HSCT at 4 time points: pre-transplantation, 14, 30 and 90- days post-transplantation. Graphs were separated by patients’ gender into two categories: **(A)** males and **(B)** females. The patient groups in each graph were categorized based on the severity of aGVHD (*triangle*- No aGVHD, *square*- with any grade aGVHD). The means are depicted as red lines with SEM. *p<0.05, **p<0.01. Mann-Whitney test.

It was reported that MIF levels could differ between males and females during disease progression (17, 48). Interestingly, we found that MIF levels were significantly elevated in male patients who developed aGVHD compared to those who did not (67 ± 56 ng/ml vs. 26 ± 10 ng/ml, p=0.009). Similar trends, with borderline significance were shown at 90 days post-HSCT (85 ± 78 ng/ml vs. 37 ± 13 ng/ml, p=0.0506) (**Figure 4A**). It is important to note that there was no difference in the percentage of male patients who developed aGVHD between patients that were transplanted from a male donor compared to male patients that were transplanted from a female donor (46.6% vs. 47.0%, p=0.918). There was no significant difference in female patients at any time point (**Figure 4B**). These findings suggest that circulating MIF in HSCT patients may contribute to disease progression and this effect is gender dependent.

## Discussion

GVHD is a major complication after HSCT. GC are the first line of treatment for both acute and chronic GVHD. However, failure to respond to GC is associated with a poor outcome with mortality rates reaching up to 80% (6, 7, 51–54). Therefore, early identification of patients who are likely to develop GVHD and those who may not respond to GC treatment [steroid-refractory (SR)] could allow for earlier intervention with alternative therapies, potentially preventing disease progression and sparing steroid toxicity. In the present study, we demonstrated that MIF levels and functional polymorphisms, are associated with GVHD severity and resistance to GC treatment.

MIF, a key mediator of numerous inflammatory diseases, was one of the first cytokines to be described (24, 25). Various studies suggested that MIF mainly contribute to diseases progression by promoting leukocyte recruitment, inhibiting apoptosis of activated monocytes, enhancing secretion of proinflammatory cytokines and counter-regulating GC suppressive effects (23, 31–33, 55).

This study evaluated the functional polymorphisms in patients undergoing HSCT, of MIF promoter and measured circulating MIF levels at several time points before and following allogeneic HSCT. A previous study by Chang et al. demonstrated that patients receiving cells from donors who carry the MIF -173C allele (which is associated with high MIF expression) had a higher risk of developing chronic GVHD, but not acute GVHD (43). In our study, both functional polymorphisms in the MIF promoter, -794 CATT5-8 repeats and -173G/C SNP, were evaluated in HSCT recipients. There was no difference in the frequencies of the -794 CATT7 and the -173 C containing genotypes between patients who did not develop aGVHD or developed only grade I aGVHD compared to those who developed grade II aGVHD. However, these genotypes were significantly associated with grades III-IV aGVHD. This suggests that high genotypic MIF expression acts as a disease modifier. Furthermore, in the multivariate analysis, this effect of MIF on disease severity was found to be superior to the effects of the conditioning regimen and HLA disparity. This role of MIF has been previously demonstrated in other autoimmune and inflammatory conditions, such as multiple sclerosis, rheumatoid arthritis and systemic sclerosis (17, 23, 27, 56). Our results are in agreement with the findings of Chang et al., as we did not observe any significant differences in the frequencies of the -173G/C genotypes between patients who did not develop aGVHD and those who did. In our study, the significant difference between the two groups was observed only for the -794 CATT polymorphism. Moreover, we have assessed MIF polymorphisms in relation to aGVHD severity and found that it was significantly correlated with the frequencies of the high-expression CATT7 containing genotypes (grade III-IV vs grade II vs grade I and non 36.8% vs. 0% vs. 5.8% p=0.0019 OR=10.4, p=0.0080 OR=10.2, respectively). These data might give clinicians an important tool for GVHD severity prediction and may help guide clinical decision-making, such as introducing additional therapeutic interventions earlier in the course of treatment for high-risk patients. Unfortunately, we did not evaluate MIF polymorphisms in donors, since most of the patients received grafts from MUD that were not available for consent. This should be further studied in the future, especially in light of the data from Toubai et al. that showed in a murine model of GVHD that GVHD severity did not differ between mice that received cells from MIF knockout (KO) mice compared with mice that received cells from WT mice. However, they reported that there was an attenuation in disease severity when the transplanted cells were from MIF KO donors compared with WT donor mice (57). Interestingly, it should be noted that in these sets of experiments, the MIF KO mice strain was only BALB/c. Therefore, it would be interesting to perform similar experiments with C57BL/6 MIF KO mice, as donor or recipient mice. This would provide an additional informative comparison where the donor and recipient mice are from the same strain.

Consistent with previous studies (12, 51, 58), approximately 30% of patients suffering from aGVHD in our study had SR-aGVHD. This underscores the need to identify biomarkers for SR GVHD. We suggest that MIF functional genetic polymorphisms could serve as potential biomarkers for this purpose. This novel finding in aGVHD is in-line with studies on asthma and inflammatory bowel disease, which have also described the effect of MIF on resistance to GC therapy (35–37). Moreover, it was demonstrated that T cells from individuals carrying high-expressing genotypes were more resistant glucocorticoid-induced apoptosis compared with low-expressing MIF genotypes (59).

It is important to note that the frequencies of the CATT7 containing genotypes in patients with grade III-IV (36.8%) and in patients with GVHD that are resistant to steroid treatment (46.6%) are higher than the frequency of these genotypes in the general Caucasian population (20-22%) (60).

Lo et al. and Toubai et al. reported that serum MIF levels were significantly elevated before the onset of aGVHD, suggesting its role in this pathology (41, 42). Similarly, we observed that circulating MIF levels were significantly elevated in patients that developed severe aGVHD compared to MIF levels before HSCT and to patients who did not develop aGVHD or developed grade I disease. This significant difference was noted on day-30 post-HSCT, before the median day of aGVHD onset, which occurred on day 36 post-HSCT, pointing to MIF’s potential role in the etiology of acute GVHD. The lack of significance on day 90 post-HSCT could be attributed to the impact of second-line anti-GVHD treatments and to the fact that some patients with severe aGVHD did not survive to this time point. Interestingly, we show that MIF serum levels were significantly elevated in males who developed GVHD but not in females. This observation aligns with previous studies that reported a gender dependent effect of MIF on multiple sclerosis progression (17). We did not observe any gender dependent effect in MIF genetic polymorphisms, which could point to gender dependent post-translational regulation on MIF levels. This effect should be further discerned in a larger study. It is important to note that while some studies on large and clinically homogeneous inflammatory syndromes have observed a concordance between MIF genotype and serum levels, other studies have not (47, 61). In patients undergoing allogeneic HSCT, MIF levels can also be influenced by donor cells, introducing a potentially significant factor that may impact the relationship between genotype and serum levels. It is also important to note that serum MIF levels may not fully represent MIF expression, as its production can be genetically regulated at the site of inflammation.

This study is limited by its relatively small cohort size and the lack of donor samples. Furthermore, since the samples were collected at predefined time points, we did not correlate them with the day of aGVHD onset or the response to treatment.

In conclusion, our findings suggest that MIF acts as a disease modifier in aGVHD. High recipient genotypic MIF expression likely corresponds with increased MIF levels and additional proinflammatory factors that enhance disease progression and resistance to GC treatment. Taken together, we propose potential use of MIF polymorphism as a tool for personalized tailoring of GVHD prophylaxis and treatment. In addition, novel therapies targeting MIF such as, ISO-1 (62), partial MHC class II constructs (63) and Ibudilast (64), could potentially reduce aGVHD severity and counteract the inhibitory effect on GC. This approach might also preserve the graft versus leukemia response and aligns with the role of MIF in inflammation progression and exacerbation rather than initiation.

## Data Availability

The raw data supporting the conclusions of this article will be made available by the authors, without undue reservation.
