# Supplementary material for: MIF functional polymorphisms are associated with acute GVHD progression and steroid-refractoriness
Source: Front Immunol. 2025 Apr 30;16:1504976. doi: 10.3389/fimmu.2025.1504976 (PMC12105047; doi:10.3389/fimmu.2025.1504976)
Supplement: Supplementary file 1 [file DataSheet1.docx]

**Supplementary Table 1: MIF promoter polymorphisms in HSCT patients.**

| **OR**  **(95% CI)** | **P value** | **aGVHD Grades II-IV (%) (n=34)** | **No aGVHD\ Grade I aGVHD**  **(%) (n=52)** | **MIF -794 CATT genotypes** |
| --- | --- | --- | --- | --- |
|  | NS | 2 (5.9%) | 6 (11.5%) | **55** |
|  | NS | 14 (41.2%) | 20 (38.5%) | **56** |
|  | NS | 1 (2.9%) | 2 (3.8%) | **57** |
|  | NS | 11 (32.4%) | 23 (44.2%) | **66** |
| 10.9 (1.2-95.3) | 0.014 | 6 (17.6%) | 1 (1.9%) | **67** |
| 4.2 (1.0-17.7) | 0.045 | 7 (20.6%) | 3 (5.8%) | **-794 CATT7 containing genotypes** |
|  |  |  |  | **MIF -173 SNP genotype** |
|  | NS | 24 (70.6%) | 36 (69.2%) | **GG** |
|  | NS | 9 (26.5%) | 16 (30.8%) | **GC** |
|  | NS | 1 (2.9%) | 0 | **CC** |
|  | NS | 10 (29.4%) | 16 (30.8%) | **-173 C containing genotypes** |

P values were calculated by Fisher exact test. OR-odds ratio. CI- confidence interval. NS- not significant

**Supplementary Table 2: MIF promoter polymorphisms in HSCT patients-disease severity.**

| **OR**  **(95% CI)** | **P value** | **Grade III + IV aGVHD**  **(N=18)**  **(%)** | **Grade II aGVHD**  **(N=16)**  **(%)** | **No aGVHD +Grade I aGVHD**  **(N=52)**  **(%)** | **MIF -794 CATT genotypes** |
| --- | --- | --- | --- | --- | --- |
|  | NS | 0 | 2 (12.5%) | 6 (11.5%) | **55** |
|  | NS | 7 (36.8%) | 7 (43.8%) | 20 (38.5%) | **56** |
|  | NS | 1 (5.3%) | 0 | 2 (3.8%) | **57** |
|  | NS | 4 (21.1%) | 7 (43.8%) | 23 (44.2%) | **66** |
| 25.5 (2.8-232.1) ^a^  8 (0.8-75.5) ^b#^ | 0.0008^a^  0.019^b^ | 6 (31.6%) | 0 | 1 (1.9%) | **67** |
| 10.4 (2.3-46.7) ^a^  10.2 (1.1-94.8) ^b#^ | 0.0019^a^  0.0080^b^ | 7 (36.8%) | 0 | 3 (5.8%) | **-794 CATT7 containing genotypes** |
|  |  |  |  |  |  |
|  |  |  |  |  | **MIF -173 SNP genotype** |
|  | NS | 9 (47.7%) | 15 (93.8%) | 36 (69.2%) | **GG** |
|  | NS | 8 (42.1%) | 1 (6.3%) | 16 (30.8%) | **GC** |
|  | NS | 1 (5.3%) | 0 | 0 | **CC** |
| 15 (1.6-138.8) ^b^ | 0.0078^b^ | 9 (47.7%) | 1 (6.3%) | 16 (30.8%) | **-173 C containing genotypes** |

P values were calculated by Fisher exact test. OR-odds ratio. CI- confidence interval. a-No GvHD vs. Grades III+ IV. b- Grade II vs. Grades III+ IV. # Halden's modification. NS- not significant.

**Supplementary Table 3: MIF promoter polymorphisms in acute GVHD patients, grades II-IV.**

| **OR**  **(95% CI)** | **P value** | **Steroid refractory (n=15) (%)** | **Steroid responsive (n=19) (%)** | **MIF -794 CATT genotypes** |
| --- | --- | --- | --- | --- |
|  | NS | 0 | 2 (10.5%) | **55** |
|  | NS | 4 (26.7%) | 10 (52.7%) | **56** |
|  | NS | 1 (6.6%) | 0 | **57** |
|  | NS | 4 (26.7%) | 7 (36.8%) | **66** |
| 12.6 (1.3-121.4) | 0.003 | 6 (40.0%) | 0 | **67** |
| 16.6 (1.7-158.0) | 0.0011 | 7 (46.6%) | 0 | **-794 CATT7 containing genotypes** |
|  |  |  |  | **MIF -173 SNP genotype** |
|  | NS | 6 (40.0%) | 18 (94.7%) | **GG** |
| 20.5 (2.1-196.1) | 0.0024 | 8 (53.3%) | 1 (5.3%) | **GC** |
|  | NS | 1 (6.7%) | 0 | **CC** |
| 27.0 (2.8-259.5) | 0.0007 | 9 (60.0%) | 1 (5.3%) | **-173 C containing genotypes** |

P values were calculated by Fisher exact test. OR-odds ratio. CI- confidence interval. NS- not significant.

**Supplementary Figure 1:** **MIF serum levels in HSCT patients.** MIF levels were evaluated in patients that received HSCT at 4 time points: pre-transplantation, 14, 30 and 90-days post-transplantation. The patient groups were categorized based on the severity of aGVHD (*triangle*-aGVHD\ grade I; *circle*-aGVHD grade II-IV). The means are depicted as red lines with SEM. *p<0.05 Mann-Whitney test.
